# Vitamin D, vitamin D supplementation and atrial fibrillation risk in the general population: updated systematic review and meta-analysis of prospective studies

**DOI:** 10.3389/fnut.2023.1246359

**Published:** 2023-09-21

**Authors:** Xiaoli Ding, Jiying Lai, Hehui Zhang, Zongwen Guo

**Affiliations:** ^1^Clinical Laboratory, The First Affiliated Hospital of Gannan Medical University, Ganzhou, Jiangxi, China; ^2^Department of Critical Care Medicine, The First Affiliated Hospital of Gannan Medical University, Ganzhou, Jiangxi, China

**Keywords:** atrial fibrillation, vitamin D, relationship, supplementation, meta

## Abstract

**Background:**

Since the association of vitamin D with atrial fibrillation (AF) risk is still unclear, we conducted this updated meta-analysis of prospective studies to identify the relationship between vitamin D or vitamin D supplementation and AF in the general population.

**Methods:**

We conducted a comprehensive search of multiple databases up to May 2023 for studies reporting vitamin D and AF. The hazard ratios (HRs) with 95% confidence intervals (CIs) were pooled by a random-effects model.

**Results:**

A total of seven studies were included in this meta-analysis. Vitamin D deficiency (<20 ng/ml) was associated with increased AF incidence (HR: 1.12, 95% CI: 1.005–1.25). The HR was not significant with vitamin D insufficiency (20–30 ng/ml; HR: 1.09, 95% CI: 0.98–1.21). Each 10 ng/ml increase in serum vitamin D was associated with a significantly decreased AF incidence (HR: 0.95, 95% CI: 0.93–0.97). Two studies reported the effect of vitamin D supplements on AF incidence but reached inconsistent results.

**Conclusions:**

Vitamin D deficiency or insufficiency was associated with an increased risk of AF in the general population. The role of vitamin D supplementation in AF prevention needs further investigation.

## Introduction

Atrial fibrillation (AF) is the leading cardiac arrhythmia and its prevalence varies concerning geography, social economy, age, sex, and ethnicity (1–3). Once established, AF is associated with several severe complications (e.g., stroke, ischaemic heart disease) (4), mortality (4), and high healthcare costs (5). In addition, even for individuals without a history of cardiovascular diseases, AF still contributes to elevated mortality (6). Accordingly, measures to prevent and/or delay AF that can be generalized on a population level are of great value but are scarce.

Vitamin D is an important nutrient that can be ingested from in series of foods (e.g., fish, sun-dried mushrooms, milk) or dietary supplements. In addition, it can be synthesized when exposed to ultraviolet rays. In recent years, vitamin D has been hypothesized to be linked with cardiovascular diseases through its effect on the inflammation or renin-angiotensin-aldosterone (RAAS) (7). Therefore, vitamin D may be one premium option for the prevention or treatment of AF if its beneficial effects on AF risks are confirmed. In recent years, numerous studies on the association between vitamin D and AF have been performed, but they reported inconsistent findings. A dose-response meta-analysis by Liu et al. (8) demonstrated that vitamin D deficiency or inadequacy increased the risk of AF by 23 or 14%, respectively. However, significant heterogeneity existed among the included studies. In addition, a null association between vitamin D and new-onset AF was reported in a former meta-analysis by Huang et al. (9). However, they did not differentiate between the new-onset and postoperative AF. Similarly, no significant association between vitamin D and AF risk was also demonstrated by two recent Mendelian randomization studies (10).

Furthermore, the Vitamin D and OmegA-3 TriaL (VITAL) Rhythm Study, a randomized, double-blind, placebo-controlled trial, did not find a causal relationship between vitamin D supplementation and AF risk over a median follow-up of 5 years (11). Based on the evidence above, the beneficial effects of vitamin D on AF reported previously may be overestimated to some extent. Therefore, we conducted this updated meta-analysis of prospective studies to identify the relationship between vitamin D or vitamin D supplementation and AF in the general population.

## Methods

### Literature search and study selection

This work was reported according to the PRISMA guidelines. Two authors (Xiaoli Ding and Jiying Lai) independently conducted a comprehensive search of multiple databases (PubMed, Embase, Cochrane Library) from inception to May 2023, using two sets of MeSH words and keywords: atrial fibrillation and vitamin D (Supplementary Table S1). Additionally, we searched ClinicalTrials.gov, International Standard Randomized Controlled Trial Number Registry, Monthly searches of the Cochrane Central Register of Controlled Trials (CENTRAL) and other relevant publications to identify potential studies. Gray literatures were identified by Google Scholar database.

For the inclusion criteria concerning the association between vitamin D and AF, the following PICOS criteria were applied: (1) Population: general population, excluding studies focused on specific diseases like diabetes or established cardiovascular diseases. (2) Exposure vs. control: high serum vitamin D vs. low serum vitamin D levels. (3) Outcomes: studies reporting the association between serum vitamin D and AF incidence. (4) Study design: randomized control trials (RCTs) and observational prospective cohorts were included.

Retrospective studies (cross-sectional, case-control, and retrospective cohort), case reports, reviews, meta-analyses, and Mendelian studies were excluded. Reports on the association between vitamin D or vitamin D supplements and AF recurrence, and AF post-operation were also excluded. Regarding the studies on vitamin D supplements and AF, all the observational studies or RCTs reporting the effect of vitamin D supplements on AF incidence in adults were included, without restrictions on population, sample size, comparison, or follow-up.

The initial search results were imported into EndNote X9.1 software. Duplicate citations were eliminated using both automated and manual inspection. Subsequently, we carefully examined the titles and abstracts of the remaining citations. Based on this preliminary screening, we reviewed the full-text reports that seemed to meet the predefined inclusion criteria.

### Data extraction

Two authors (Xiaoli Ding and Jiying Lai) independently extracted data from the included studies, and any discrepancies were resolved through discussion. The extracted information included the first author, country, publication year, gender, mean or age, study design, population, source of population, sample size, vitamin D level, measurements of vitamin D and AF, hazard ratios (HRs) with 95% confidence intervals (CIs), and adjustments.

### Statistical analysis

Two authors (Xiaoli Ding and Jiying Lai) independently conducted searches for eligible studies, extracted data, assessed study quality, and synthesized the data. Disagreements were discussed by consent or advised by a third author (Zongwen Guo). To pool the HRs were pooled by random-effects models. Both vitamin D levels were analyzed as both a category and a continuous variable. In the category analysis, we examined the association between vitamin D deficiency and insufficiency compared to normal vitamin D levels. In the continuous analysis, study-specific slopes (vitamin D per 10 ng/ml increment) and 95% CIs were calculated from the natural logs of the reported HRs and CIs across different vitamin D categories (12). Considering the substantial difference in study design and quality evidence between observational studies and RCT trials, the results of vitamin D supplements and AF incidence were analyzed separately based on study design. Statistical heterogeneity between studies was assessed using the Cochran *Q*-test and tau2 statistics, while inconsistency across studies was measured using *I*^2^. Pre-defined subgroup analysis was conducted based on age, sex, population with vitamin D insufficiency or deficiency, region, and follow-up duration (when the number of studies exceeded five). Sensitive analyses by fixed model, excluding studies with shorter duration of follow-up (<5 years), reports without body mass index, physical activity, smoking, and hypertension were conducted.

The quality of observational studies and randomized controlled trials (RCTs) was assessed using the Newcastle-Ottawa Quality Assessment Scale (NOS) and Cochrane Bias Risk Assessment Tool, respectively (13–15). The NOS evaluates bias across three domains (selection, comparability, and outcomes) using eight items, with a maximum total score of nine. A NOS score of seven or higher indicates high quality. The Cochrane Bias tool assesses bias across seven domains, including selection bias, implementation bias, measurement bias, follow-up bias, reporting bias, and other biases. The publication bias was not assessed due to the limited number of studies (*N* < 10).

All the statistical analyses were done by using Review Manager (RevMan) version 5.4 (The Cochrane Collaboration 2020; Nordic Cochrane Center Copenhagen, Denmark). All statistical tests were double-sided, and *P* < 0.05 was considered statistically significant.

## Results

### Study selection

**Figure 1:**
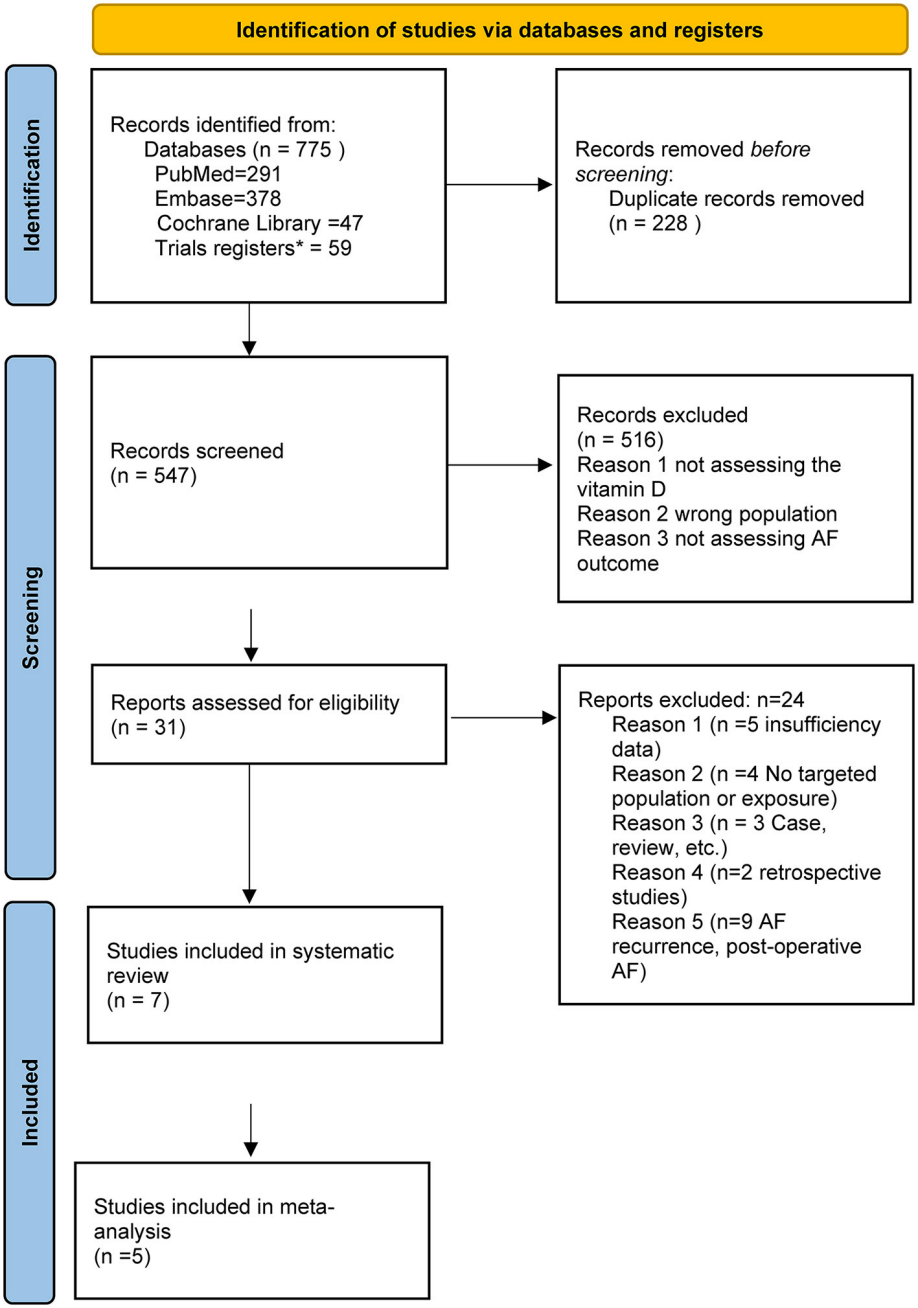
The flow diagram for this systematic review which included searches of databases.

The study selection is shown in Figure 1. Initially, a comprehensive search was conducted across multiple databases, including PubMed, Embase, and Cochrane Library and trial registers, resulting in a total of 775 records. After removing duplicate records (*n* = 228), the remaining 547 records were screened based on predetermined inclusion and exclusion criteria. Next, the remaining 31 reports were assessed for eligibility in detail in full text. Among these reports, a further review was made, resulting in the exclusion of studies (*n* = 24) based on the following reasons: (1) insufficiency data, *n* =5; (2) No targeted population or exposure, *n* = 4; (3) Case, case-serial, review, and meta-analysis, *n* = 3; (4) Retrospective studies, such as case-control studies, *n* = 2; (5) Inappropriate outcomes (AF recurrence, post-operative AF, *n* = 9; Supplementary Table S2). Finally, a total of seven studies were included in this systematic review and meta-analysis (11, 16–21). Five studies (six prospective cohorts) reported the serum vitamin D and AF incidence, whereas two studies (one RCT and one nest-case control) reported the effect of vitamin D supplements on AF incidence.

### Study characteristics and quality

The study characteristics are shown in Table 1. Overall, these studies were published between 2015 and 2022. The sample sizes ranged from 845 to 12,577. Five studies were from the United States, one was from the Netherlands, and one was from Italy. All the study individuals were community-based general population and adjusted their results by multivariable analysis in the association between vitamin D and AF. All of the studies were scored as low bias (Supplementary Table S3 and Supplementary Figure S1).

**Table 1:** Basic characteristics of the included studies.

**Section 1: Vitamin D supplements and AF**									
**Reference, country**	**Design, follow up**	**Population**	**Intervention**	**Sample size**	**Measurement of AF and vitamin D**	**Mean age (years), male (%)**	**Control**	**HR (95%CI) vitamin D supplements and AF**	**Adjustments**
Albert et al. (11), United States	RCT, 5.3 years	Over 50 without prior cardiovascular disease, cancer, or AF	vitamin D_3_ (2,000 IU/day)	12,577	Medical record, NA	66.7, 49.2	Placebo	1.09 (0.96–1.25)	None
Acharya et al. (21), United States	Nested case-control	Veterans	Vitamin D supplement for < 6 months	39,845	ICD, NA	57.6,70.0	None	Untreated level ≤ 20 ng/ml (ref) Treated level ≥30 ng/ml: 0.84 (0.73–0.95) Treated level 21–29 ng/ml: 0.89 (0.80–0.98)	Propensity score for age, gender, BMI, hypertension, DM, coronary artery disease, congestive HF, cardiomyopathy, PAD, COPD, obstructive sleep apnea, infective endocarditis, rheumatic heart disease, valvular heart disease, hyperthyroidism, depression, smoking, concomitant therapies, and the LDL-C
**Section 2: Serum vitamin D and AF incidence**									
**References, country**	**Design, follow up**	**Population**	**Source of individuals**	**Cases/** ***N***	**Measurement of AF and vitamin D**	**Mean age (years), male (%)**	**Category of vitamin D**	**Effect size (95%CI) vitamin D level and AF**	**Adjustments**
Rienstra et al. (16), United States	Prospective cohort, 9.9 years	Community-based general population	Framingham Heart Study	425/2,930	ECG, competitive protein-binding assay and radioimmunoassay	65, 44	Per 1 ng/mL	0.99 (0.89–1.10)	Age, sex, BMI, PR interval, and cardiac murmur
Mathew et al. (17), United States	Prospective cohort, 7.7 years	Community-based general population	MESA Study	291/6,398	ECG, hospital discharge diagnoses, inpatient and outpatient physician claims data	62, 53.5	Per 10 ng/mL	0.92 (0.81–1.03)	Age, sex, race, study site, attained education, low density, lipid-lowering medications, current smoking, DM, physical activity, height, height squared, weight, urine albumin-creatinine-ratio, eGFR, systolic blood pressure, and use of hypertension medication
	Prospective cohort, 8.0 years	Community-based general population	CHS Study	229/1,350		77, 71.3	Per 10 ng/ml	1.00 (0.88–1.14)	
Vitezova et al. (20), Netherlands	Prospective cohort, 12 years	Community-based general population	Rotterdam Study	263/3,295	ECG, electrochemiluminescence immunoassay	71, 41	< 50 nmol/L 50–74 nmol/L ≥75 nmol/L	Ref 0.82 (0.60–1.11) 0.76 (0.52–1.12)	Age, sex, income, education, BMI, physical activity, diet quality score, smoking status and season and year when the blood was drawn
Alonso et al. (18), United States	Prospective cohort, 18 years	Community-based general population	ARIC study	1,866/12,303	ECG, high-sensitivity mass spectrometer	57, 43	< 20 ng/ml 21–29 ng/ml ≥30 ng/ml	1.10 (0.96–1.26) 1.09 (0.97–1.22) ref	Age, sex, race, study center, education, alcohol consumption, height, BMI, smoking status, physical activity, systolic and diastolic BPs, medication, DM, coronary heart disease, HF, hsCRP, NT-pro-BNP, and eGFR
Trevisan et al. (19), Italy	Prospective cohort, 4.2	Community-based general population with elderly	Progetto Veneto Anziani cohort	134/2,418	ICD-9, use of anti-coagulant drugs and antiarrhythmic drugs, radioimmunoassay	76.0,40.1	< 20 ng/ml ≥30 ng/ml	1.27 (0.84–1.94) Ref	Age, sex, BMI, Short Physical Performance Battery total score, GFR, serum calcium levels, season, educational level, smoking, drinking, hypertension, cardiovascular diseases, use of lipid lowering drugs, PTH levels

AF, atrial fibrillation; HF, heart failure; hsCRP, high-sensitivity C-reactive protein; LAD, left atrium diameter; LAVI, left atrial volume index; LV, left ventricle; LVEF, left ventricular ejection fraction; PASP, pulmonary artery systolic pressure; BMI, body mass index; BP, blood pressure; DM, diabetes mellitus; NT-proBNP, N-terminal of the prohorme B-type natriuretic peptide; eGFR, estimated glomerular filtration rate; HDL-C, high-density lipoprotein cholesterol; LDL-C, low-density lipoprotein cholesterol; COPD, chronic obstructive pulmonary disease; RAS, renin-angiotensin system; ECG: electrocardiography: MESA: Multi-Ethnic Study of Atherosclerosis; CHS: Cardiovascular Health Study; ICD, International classification of diseases; ACEI, Angiotensin-Converting Enzyme Inhibitors; ARB, angiotensin receptor blocker; MS/MS, Cascade mass spectrometry; DHA, docosahexaenoic acid; HCUP, Healthcare Research and Quality's Healthcare Cost and Utilization Project.

### Serum vitamin D on AF incidence

**Figure 2:**
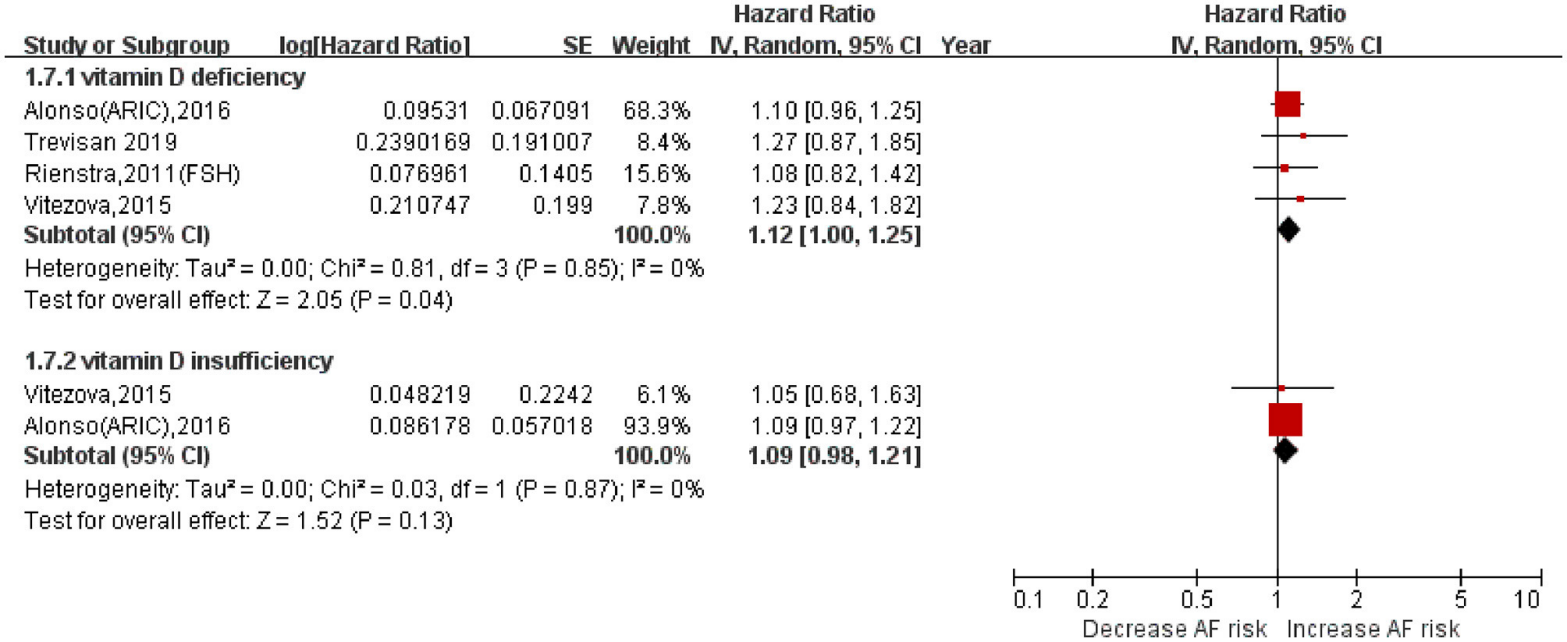
Association of serum vitamin D (deficiency or insufficiency) with AF incidence.

Six prospective cohorts with 2,917 cases/28,694 participants were included (16, 18, 20, 22–24). As shown in Figure 2, vitamin D deficiency (<20 ng/ml) was associated with increased AF incidence (HR: 1.12, 95% CI: 1.005–1.25; *I*^2^ = 0%). The HR was not significant with vitamin D insufficiency (20–30 ng/ml; HR: 1.09, 95% CI: 0.98–1.21; *I*^2^ = 0%).

**Figure 3:**
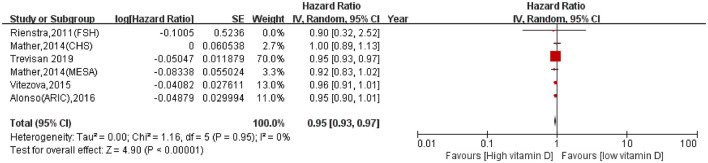
Association of each 10 ng/ml increase in serum vitamin D with AF incidence.

When vitamin D was analyzed as a continuous variable, each 10 ng/ml increase in serum vitamin D was associated with a significantly decreased AF incidence (HR: 0.95, 95% CI: 0.93–0.97, *I*^2^ = 0%; Figure 3). Excluding the study with the largest weight did not significantly change the results (HR: 0.96, 95% CI: 0.92–0.99, *I*^2^ = 0%).

### Vitamin D supplement and AF incidence

An RCT showed randomization to vitamin D_3_ (2,000 IU/day, *n* = 6,272) treatments for who aged >50 years did not significantly reduce the incidence of AF compared with placebo after a median follow-up of more than 5 years (HR: 1.09; 95% CI, 0.96–1.25; *P* = 0.19) (11). Conversely, a nested case-control study using propensity score matching demonstrated that vitamin D treatment maintaining levels above 20 ng/ml for at least 6 months was associated with a significantly lower incidence of new-onset AF compared to untreated patients whose levels remained at or below 20 ng/ml (21) (vitamin D treated, level 21 to 29 ng/ml, HR: 0.89, 95% CI: 0.80–0.98; vitamin D treated, level ≥30 ng/ml, HR: 0.84, 95% CI: 0.73–0.0.95).

### Subgroup analyses and sensitive analyses

Subgroup analyses according to sample size, AF cases, mean age, and region did not find evidence of interactions (all *P* > 0.05). Sensitive analyses by fixed model, excluding studies with shorter duration of follow-up (<5 years), reports without body mass index, physical activity, smoking, and hypertension generated confirmed results (Table 2).

**Table 2:** Subgroup and sensitivity analyses-serum vitamin D and atrial fibrillation incidence, per 10 ng/ml increase.

**Items**		**Number of cohorts**	**HR (95%CI)**	**P-value**	P-value${}_{h\left(\%\right)}^{*}$	**P-value${}_{h\left(\%\right)}^{\text{\#}}$**
Result of primary analysis		6	0.95 [0.93, 0.97]	< 0.001	0	
Age	< 65 years	3	0.94 [0.89, 0.9975]	0.04	0	0.73
	>65 years	3	0.95 [0.93, 0.97]	< 0.001	0	
Sample size	< 3,000	3	0.96 [0.92, 0.99]	0.01	0	0.98
	≥3,000	3	0.95 [0.93, 0.97]	< 0.001	0	
AF case	< 200	5	0.95 [0.93, 0.97]	0.01	0	0.79
	≥200	1	0.96 [0.92, 0.99]	< 0.001	0	
Region	European	3	0.95 [0.93, 0.97]	< 0.001	0	0.96
	America	3	0.95 [0.91, 0.995]	0.049	0	
Fixed model		5	0.95 [0.93, 0.97]	< 0.001	0	
Exclusion of subjects	Follow-up < 5 years	5	0.96 [0.92, 0.99]	0.01	0	
	Not adjusted PA excluded	5	0.95 [0.93, 0.97]	< 0.001	0	–
	Not adjusted DM excluded	3	0.95 [0.91, 0.9996]	0.049	0	–
	Not adjusted HTN excluded	4	0.95 [0.93, 0.97]	< 0.001	0	–
	Not adjusted smoking excluded	5	0.95 [0.93, 0.97]	< 0.001	0	–

^*^P-value for within-group heterogeneity.

^#^P-value for subgroup difference.

## Discussion

Our current meta-analysis revealed that vitamin D deficiency but not insufficiency was associated an increased risk of AF in the general population. In addition, an increment of 10 ng/ml of vitamin D was associated with a tiny decrease in AF risk.

AF is the most common supraventricular arrhythmia with complex pathogenesis. To date, although the underlying pathogenesis of AF has not been fully understood, the disorders of the RAAS and the inflammation involvement are two key factors. Vitamin D, as an exquisite fundamental micronutrient for humans, not only plays an indispensable role in maintaining calcium-phosphate homeostasis and musculoskeletal metabolism (25) but also in the modulation of inflammatory response and RAAS activity (26). In addition, Hanafy et al. and coworkers confirmed that vitamin D can prevent or terminate the occurrence of AF through direct electromechanical effects on the left atrium of the heart failure rabbit (27). Therefore, it may be theoretically reasonable to speculate the protective effects of vitamin D on AF. However, this speculation was not fully justified in clinical practice.

To date, a number of studies investigating the association between vitamin D and AF risk have been reported but with inconclusive findings. Results from observational studies have revealed that patients with vitamin D deficiency were associated with a higher risk of AF compared with patients with normal levels (24, 28–30). Nevertheless, such an association was not identified in the prospective studies (16–18, 20). By only pooling relevant prospective studies conducted in the general population, we found that the AF risk marginally increased for individuals with vitamin D deficiency when compared with those with normal vitamin D levels. In addition, per 10 ng/ml increase in vitamin D is associated with only a 5% decrease in AF risk. In contrast, by performing a Mendelian randomization analysis using the summary statistics obtained for single-nucleotide polymorphisms (SNPs) identified from genome-wide association meta-analyses, Yang et al. (31) found no causal relationship between vitamin D levels and AF risk. Their findings were also collaborated by another relevant Mendelian randomization analysis by Zhang et al. (32). Accordingly, it is suggested that certain protective effects of vitamin D on AF occurrence may exist, but considering vitamin D as a therapeutic target to delay AF progress in clinical settings may not be recommended.

The mechanism of vitamin D deficiency on AF development is not fully understood. Murdaca et al. (33) have previously concluded that vitamin D deficiency plays a role in the pathophysiology of several autoimmune diseases such as psoriasis vulgaris, iridocyclitis, ulcerative colitis, thyrotoxicosis, and Crohn's disease. Vitamin D deficiency may alter the microbiome by changing the composition of the microbiome and the integrity of the intestinal epithelial barrier, or affect the immune system primarily through the vitamin D receptor (34). The IL31/IL33 axis plays an important role in the development of these diseases (35). It is possible that vitamin D deficiency may impair the IL31/IL33 axis favoring myocardial inflammation and AF development, which needs further investigation.

To further explore the potential benefits of vitamin D supplementation on the prevention and delay of AF, several clinical trials have been performed in clinical settings. The Women's Health Initiative (WHI) calcium and vitamin D (CaD) trial, a randomized, double-blind trial, investigated the effect of supplementation of CaD supplements in the primary prevention of AF in postmenopausal women. In this trial, postmenopausal women were randomized to either receive CaD supplements (400 IU/day of vitamin D3 and 1,000 mg/day of elemental calcium) or a placebo, and no significant difference in the incident AF rates between the two groups was observed (36). However, the study did not distinguish the independent effect of vitamin D supplementation from calcium supplementation. In addition, since previous studies reported an increased risk of cardiovascular events with calcium supplementation regardless of whether vitamin D supplementation or not (37, 38), it is possible that the increased AF risk resulting from calcium supplementation counterbalances the benefits of vitamin D supplementation to some extent. In another randomized double-blind placebo-controlled trial, Albert et al. (11) also did not find a causal relationship between vitamin D supplementation (2,000 IU/day of vitamin D) and AF risk over a median follow-up of 5 years. Nevertheless, only 12.7% of the participants in that trial had baseline vitamin D deficiency, possibly leading to the limited power to detect the potential benefits of vitamin D supplementation for this subset. In contrast, the null benefits of vitamin D supplementation and incident AF risk reported by the former two studies were not been collaborated by a recent nested case-control study (21). In the study, 39,845 participants with baseline vitamin D ≤ 20 ng/ml were included and grouped into three groups: group-A (untreated), group-B (treated, levels 21–29 ng/ml), and group-C (treated, levels ≥30 ng/ml). After being treated for ≥6 months, the AF risk was lower in group B and group C when compared with group A, and the AF risk was not significantly different between group B and C for the whole included individuals. However, for men >65 years with hypertension or diabetes mellitus, an added benefit was further acquired when the vitamin D was ≥30 ng/ml. Nevertheless, due to the observational design of the study, the findings may be confounded by the additional supplementation that was not recorded in the medical records.

## Implications and future directions

Although not all the previous studies have indicated vitamin D deficiency as an independent risk factor for AF, most of the current evidence supports the potential associations of vitamin D deficiency with AF. Of note, analyzing patients with comorbidities such as heart failure and diabetes mellitus might limit the findings between vitamin D deficiency and AF because these comorbidities could increase AF occurrence regardless of vitamin D levels. Therefore, we only included prospective studies focusing on the general population, and found that vitamin D deficiency was associated with an increased risk of AF. Nevertheless, due to the observational nature of studies and several uncontrolled factors (e.g., parathyroid hormone, calcium level, and seasonal, sex, and ethnic variations in vitamin D levels), the causal relationship between vitamin D and AF risk was still inconclusive. Further prospective, randomized, double-blinded, large clinical trials in this field would be needed since they have advantages over observational studies. Although our data provided some evidence as to the importance of considering vitamin D supplementation for AF, there was still no recommendation of supplementation in clinical practice before the robust evidence.

## Limitations

Several limitations should be considered in this meta-analysis. First, the protocol of this systematic review and meta-analysis was not registered in the PROSPERO (International Platform of Registered Systematic Review and Meta-analysis Protocols). Second, due to the inherent limitation of observational data, the risk of bias or unexpected confounders could not be excluded. Our findings did not suggest a causal relationship between vitamin D levels and AF risks. Third, vitamin D levels could be affected by several factors (e.g., parathyroid hormone, calcium level, lifestyle, diet, and seasonal variation), which limits the findings and needs to be controlled in well-designed randomized trials. Finally, further investigations of the benefits of vitamin D supplementation on incident AF are still warranted due to the limited number of studies. In addition, the treatment dose and duration of vitamin D supplementation, baseline vitamin D levels, and targeted population should be carefully considered.

## Conclusions

Vitamin D deficiency but not insufficiency was associated an increased risk of AF in the general population. The role of vitamin D supplementation in AF prevention needs further investigation.

## Data availability statement

The original contributions presented in the study are included in the article/Supplementary material, further inquiries can be directed to the corresponding author.

## Author contributions

All authors listed have made a substantial, direct, and intellectual contribution to the work and approved it for publication.
