# Supplementary material for: Vitamin D, vitamin D supplementation and atrial fibrillation risk in the general population: updated systematic review and meta-analysis of prospective studies
Source: Front Nutr. 2023 Sep 21;10:1246359. doi: 10.3389/fnut.2023.1246359 (PMC10551443; doi:10.3389/fnut.2023.1246359)
Supplement: Supplementary file 1 [file Data_Sheet_1.PDF]

Supplemental Material

Vitamin D, vitamin D supplementation and atrial fibrillation risk in the general population: updated systematic review and meta-analysis of prospective studies

Running title: Vitamin D, vitamin D supplementation and AF

Xiaoli Ding[1], Jiying Lai[2], Hehui Zhang[2], Zongwen Guo[2]

1. Clinical Laboratory, The First Affiliated Hospital of Gannan Medical University, Ganzhou, Jiangxi Province, China.

2. Department of Critical Care Medicine, The First Affiliated Hospital of Gannan Medical University, Ganzhou, Jiangxi Province, China.

First author: Xiaoli Ding

Corresponding author:

Zongwen Guo Email: zongwen_guo@163.com.

Supplementary Table 1: Search strategy

PubMed database

Search	Query
#1	atrial fibrillation
#2	atrial flutter
#3	arrhythmia
#4	vitamin D
#5	25(OH)D
#6	Cholecalciferol
#7	25-hydroxyvitamin D3
#8	#1 OR #2 OR #3
#9	#4 OR #5 OR #6 OR #7
#10	#8 AND #9

Embase database

Search	Query
#1	atrial fibrillation
#2	atrial flutter
#3	arrhythmia
#4	vitamin D
#5	25(OH)D
#6	Cholecalciferol
#7	25-hydroxyvitamin D3
#8	#1 OR #2 OR #3
#9	#4 OR #5 OR #6 OR #7
#10	#8 AND #9

Cochrane library

Search	Query
#1	atrial fibrillation
#2	atrial flutter
#3	arrhythmia
#4	vitamin D
#5	25(OH)D
#6	Cholecalciferol
#7	25-hydroxyvitamin D3
#8	#1 OR #2 OR #3
#9	#4 OR #5 OR #6 OR #7
#10	#8 AND #9

Supplementary Table 2. Studies excluded(n=24) with reasons

Reasons (according to PICOS)		Studies excluded (n=23)
Reason 1<br>n = 5 insufficiency data	1.	Qayyum F, Landex NL, Agner BR, Rasmussen M, Jøns C, Dixen U. Vitamin D deficiency is unrelated to type of atrial fibrillation and its complications. Dan Med J. 2012 Sep;59(9):A4505. PMID: 22951200.
	2.	Turkkolu ST, Selçuk E, Köksal C. Biochemical predictors of postoperative atrial fibrillation following cardiac surgery. BMC Cardiovasc Disord. 2021 Apr 9;21(1):167.
	3.	Çakır OM. Low vitamin D levels predict left atrial thrombus in nonvalvular atrial fibrillation. Nutr Metab Cardiovasc Dis. 2020 Jun 25;30(7):1152-1160.
	4.	Barsan M, Brata AM, Ismaiel A, Dumitrascu DI, Badulescu AV, Duse TA, Dascalescu S, Popa SL, Grad S, Muresan L, Maerescu CM, Cismaru G, Brata VD. The Pathogenesis of Cardiac Arrhythmias in Vitamin D Deficiency. Biomedicines. 2022 May 26;10(6):1239.
	5.	Anees MA, Ahmad MI, Chevli PA, Li Y, Soliman EZ. Association of vitamin D deficiency with electrocardiographic markers of left atrial abnormalities. Ann Noninvasive Electrocardiol. 2019 May;24(3):e12626.
Reason 2<br>N=4 No targeted population or exposure	6.	Chen WR, Liu ZY, Shi Y, Yin DW, Wang H, Sha Y, Chen YD. Relation of low vitamin D to nonvalvular persistent atrial fibrillation in Chinese patients. Ann Noninvasive Electrocardiol. 2014 Mar;19(2):166-73. doi: 10.1111/anec.12105. Epub 2013 Nov 8. PMID: 24206389; PMCID: PMC6932220.

	7. Ozcan OU, Gurlek A, Gursoy E, Gerede DM, Erol C. Relation of vitamin D deficiency and new-onset atrial fibrillation among hypertensive patients. J Am Soc Hypertens. 2015 Apr;9(4):307-12.<br>8. Boursiquot BC, Larson JC, Shalash OA, Vitolins MZ, Soliman EZ, Perez MV. Vitamin D with calcium supplementation and risk of atrial fibrillation in postmenopausal women. Am Heart J. 2019 Mar;209:68-78.<br>9. Belen E, Aykan AC, Kalaycioglu E, Sungur MA, Sungur A, Cetin M. Low-Level Vitamin D Is Associated with Atrial Fibrillation in Patients with Chronic Heart Failure. Adv Clin Exp Med. 2016 Jan-Feb;25(1):51-7.
Reason 3<br>n =3 Case, review, and meta-analyses	10. Zhang Z, Yang Y, Ng CY, Wang D, Wang J, Li G, Liu T. Meta-analysis of Vitamin D Deficiency and Risk of Atrial Fibrillation. Clin Cardiol. 2016 Sep;39(9):537-43.<br>11. Huang WL, Yang J, Yang J, Wang HB, Yang CJ, Yang Y. Vitamin D and new-onset atrial fibrillation: A meta-analysis of randomized controlled trials. Hellenic J Cardiol. 2018 Mar-Apr;59(2):72-77.<br>12. Cosentino N, Campodonico J, Milazzo V, De Metrio M, Brambilla M, Camera M, Marenzi G. Vitamin D and Cardiovascular Disease: Current Evidence and Future Perspectives. Nutrients. 2021 Oct 14;13(10):3603.
Reason 4<br>n = 2 retrospective studies	13. Patel U, Yousuf S, Lakhani K, Raval P, Kaur N, Okafor T, Shah C, Singh H, Martin M, Nwodika C, Yogarajah A, Rakholiya J, Patel M, Chakinala RC, Shah S. Prevalence and Outcomes Associated with Vitamin D Deficiency among Indexed Hospitalizations with Cardiovascular Disease and Cerebrovascular Disorder-A

	Nationwide Study. Medicines (Basel). 2020 Nov 22;7(11):72.<br>14. Demir M, Uyan U, Melek M. The effects of vitamin D deficiency on atrial fibrillation. Clin Appl Thromb Hemost. 2014 Jan;20(1):98-103.
Reason 5<br>n = 9 AF recurrence, post-operative AF	15. Cerit L, Özcem B, Cerit Z, Duygu H. Preventive Effect of Preoperative Vitamin D Supplementation on Postoperative Atrial Fibrillation. Braz J Cardiovasc Surg. 2018 Jul-Aug;33(4):347-352.<br>16. Shadvar K, Ramezani F, Sanaie S, Maleki TE, Arbat BK, Nagipour B. Relationship between plasma level of vitamin D and post operative atrial fibrillation in patients undergoing CABG. Pak J Med Sci. 2016 Jul-Aug;32(4):900-4.<br>17. Gode S, Aksu T, Demirel A, Sunbul M, Gul M, Bakır I, Yeniterzi M. Effect of vitamin D deficiency on the development of postoperative atrial fibrillation in coronary artery bypass patients. J Cardiovasc Thorac Res. 2016;8(4):140-146.<br>18. Özsin KK, Sanrı US, Toktaş F, Kahraman N, Yavuz Ş. Effect of Plasma Level of Vitamin D on Postoperative Atrial Fibrillation in Patients Undergoing Isolated Coronary Artery Bypass Grafting. Braz J Cardiovasc Surg. 2018 May-Jun;33(3):217-223.<br>19. Skuladottir GV, Cohen A, Arnar DO, Hougaard DM, Torfason B, Palsson R, Indridason OS. Plasma 25-hydroxyvitamin D2 and D3 levels and incidence of postoperative atrial fibrillation. J Nutr Sci. 2016 Feb 15;5:e10.<br>20. Canpolat U, Aytemir K, Hazirolan T, Özer N, Oto A. Relationship between vitamin D level and left atrial fibrosis in patients with lone paroxysmal atrial fibrillation undergoing cryoballoon-based catheter ablation. J Cardiol. 2017 Jan;69(1):16-23.

21. Emren SV, Aldemir M, Ada F. Does Deficiency of Vitamin D Increase New Onset Atrial Fibrillation after Coronary Artery Bypass Grafting Surgery? Heart Surg Forum. 2016 Aug 22;19(4):E180-4.
22. Yang S, Zhi H, Sun Y, Wang L. Circulating Vitamin D Levels and the Risk of Atrial Fibrillation: A Two-Sample Mendelian Randomization Study. Front Nutr. 2022 Mar 28;9:837207.
23. Zhang N, Wang Y, Chen Z, Liu D, Tse G, Korantzopoulos P, Letsas KP, Goudis CA, Lip GYH, Li G, Zhang Z, Liu T. Circulating Vitamin D Concentrations and Risk of Atrial Fibrillation: A Mendelian Randomization Study Using Non-deficient Range Summary Statistics. Front Nutr. 2022 Jun 17;9:842392.

Supplementary Table 3. Quality assessment of cohort studies

First author (Publication Year)	Newcastle-Ottawa Scale									
	Selection			Comparability			Outcome			Total
	a	b	c	d	e	f	g	h	i	
Alonso, 2016	*	*	*	*	*	*	*	*		8
Rienstra, 2011	*	*	*	*	*	*	*	*		8
Vitezova, 2015	*	*	*	*	*	*	*	*		8
Mathew,2014	*	*	*	*	*	*	*	*		8
Trevisan, 2019	*	*	*	*	*	*	*			7
Acharya,2022	*	*	*	*	*	*	*	*		7

Comparability: Age = *, Other control factors = *; Adequacy of follow-up:>5 years, Follow-up rate≥80% = * for cohort studies

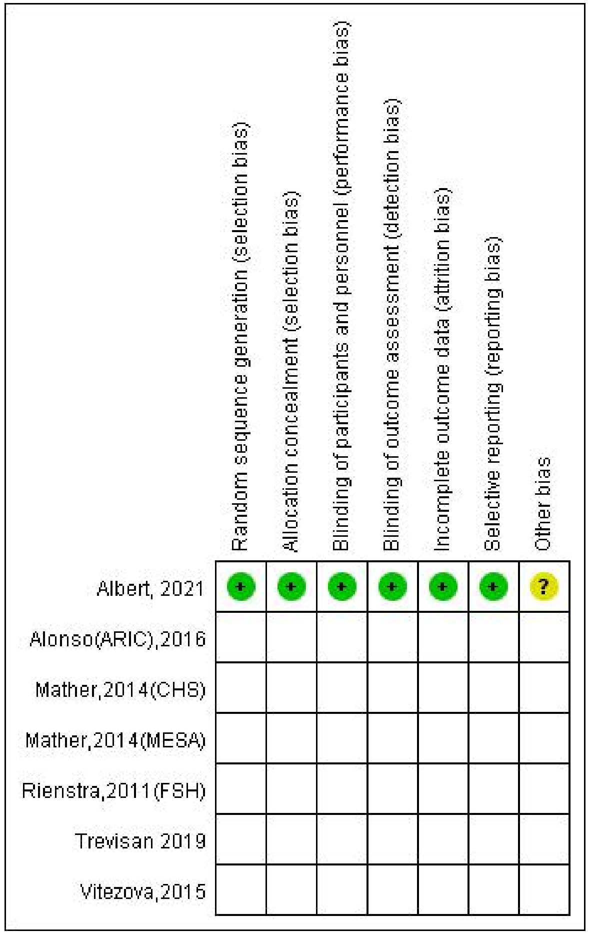

Supplementary Figure 1:Risk of bias summary: review authors' judgements about each risk of bias item for each included s
